# Shear wave elastography as a marker of anisotropy in denervated muscle tissue

**DOI:** 10.1016/j.cnp.2025.02.007

**Published:** 2025-03-05

**Authors:** Olli Kutvonen, Sari-Leena Himanen, Katri Mäkelä

**Affiliations:** a Department of Clinical Neurophysiology, Tampere University Hospital, Tampere, Finland; b Faculty of Medicine and Health Technology, Tampere University, Tampere, Finland

**Keywords:** Shear wave elastography, Anisotropy, Myography, Denervation, Heckmatt grading scale

## Abstract

**Objectives:**

To assess the capability of shear wave elastography (SWE) to detect muscle denervation.

**Methods:**

36 patients underwent electrodiagnostic studies (EDX) of the lower limbs and volunteered to undergo ultrasound examination of the Tibialis anterior (TA) and the Gastrocnemius medialis (GCM) muscles. A variable reflecting the level of anisotropy was created by calculating the difference between the longitudinal and transverse shear wave velocity (SWE-D).

**Results:**

In the TA muscles, SWE-D correlated negatively with the quantity of fibrillation potentials (FP) and the degree of interference pattern (IP) reduction (p = 0.032, r = -0.185 and p = 0.006, r = -0.236, respectively). In the GCM muscles, SWE-D only correlated with the amount of IP reduction among patients of normal weight (p = 0.030, r = -0.285). There was also a significant difference in the overall SWE-D values in the GCM muscles between patients of normal weight and obese patients (p = 0.007).

**Conclusions:**

Loss of anisotropy caused by denervation of muscle tissue may be measured quantitatively by calculating the differences between longitudinal and transverse shear wave velocities. However, obesity seems to hinder the SWE-based assessment of muscle denervation.

**Significance:**

Being able to measure anisotropy caused by denervation acts as a base for further development of SWE methods to evaluate neurogenic injury.

## Highlights

• This is a study to assess the capability of shear wave elastography to detect muscle denervation.
• A variable representing the anisotropy was calculated comparing shear wave velocities from two transducer orientations.
• Loss of anisotropy related to muscle denervation may be assessed with shear wave elastography.

## 1 Introduction

After a neurogenic injury, the histology and normal architecture of muscle tissue is altered. In denervated muscle, there is first an increase in collagen content within the endomysium and tissue fibrosis (Gonzalez et al., 2017, Tews et al., 1994) and eventually replacement of myofibers with fat cells (Tews et al., 1994, Joe et al., 2010, Ehmsen and Ahmet, 2020). Consequently, in high resolution ultrasound (HRUS) B-mode, the echogenicity of the muscle is increased. The semi-quantitative Heckmatt grading scale has been used to assess muscle pathology and its severity by estimating muscle echogenicity (Kenis-Coskun et al., 2021, Heckmatt et al., 1982). Electrodiagnostic study (EDX) consisting of nerve conduction studies (NCS) and needle electromyography (EMG) is a well-established method of examining the neuromuscular system. With EMG, it is possible to diagnose acute and chronic neurogenic lesions in muscles (Kimura, 2013, Dumitru et al., 2001). In both current clinical diagnostic routines, the interpretation of the findings partly relies on the subjective assessment of the operator.

One of the latest enhancements to HRUS studies is shear wave elastography (SWE) (Hobson-Webb, 2020). SWE uses the acoustic radiation of focused ultrasound beams to generate shear waves. Quantitative measurement of elasticity parameters can be achieved by observing the speed of propagation of the shear waves with high frame rate ultrasound imaging (Ryu and Jeong, 2017). An estimate of tissue stiffness (μ) can be calculated from the shear wave velocity (SWV, v) and tissue density (ρ) as μ = ρv^2^ (Sigrist et al., 2017). For the equitation, several assumptions of tissue behavior are expected. For example, the tissue is expected to be linear, isotropic, incompressible and unstressed (Sigrist et al., 2017, Crutison et al., 2022).

There are also several variables that can affect the acquired SWV values. These include transducer orientation, surrounding tissues, the device used, and size of the region of interest (ROI) (Schrier et al., 2020, Alfuraih et al., 2017, Miyamoto et al., 2015). Furthermore, in anisotropic tissue, such as muscle, the orientation of the ultrasound transducer plays a key role, as the properties of the material (i.e. stiffness) are different along and across the muscle fibers (Alfuraih et al., 2017, Eby et al., 2013, Aubry et al., 2013). In general, shear waves propagate faster in stiff tissues compared to soft tissues (Gennisson et al., 2010). Because muscle tissue is stiffer along the muscle fibers than across, this difference is reflected in the fast shear wave velocities measured with longitudinal transducer orientation compared to transverse orientation (Eby et al., 2013). Even a small rotation of the transducer in relation to muscle fibers can change the acquired SWV values. This is especially critical when imaging muscles that have lost their normal architecture.

In SWE, compared to healthy muscle, fibrosis has been shown to correlate with increased SWV, as it is a stiffer tissue type (Liu et al., 2015). In contrast, increased fat content in muscle has been shown to correlate with decreased SWV (Rosskopf et al., 2016). Thus, since the histopathology of the muscle changes in relation to time after the occurrence of denervation, the measured SWV may be above or below a normal reference value, depending on the moment of the study. However, both fibrosis and increased fat content make muscles more isotropic, i.e., the unidirectional fiber quality of muscle fibers is altered. Interestingly, it has been speculated that loss of normal muscle architecture and change of muscle anisotropy towards isotropy could result in convergence of parallel and transverse SWV measurements (Daube et al., 2015).

The aim of our study was to examine the association between SWE and EDX findings and the Heckmatt grading scale in Tibialis anterior (TA) and Gastrocnemius medialis (GCM) muscles. We created a SWE parameter by calculating the difference between longitudinal ad transverse SWV. We hypothesized that the loss of anisotropy in denervated muscle could be observed by a reduction of the difference between these two measurements. This new parameter might objectively detect changes in muscle tissue related to denervation through the loss of anisotropy instead of changes of stiffness as such. The benefit of basing the evaluation on the loss of isotropy would be the exclusion of possible misleading time related histopathological changes of variable fat and connective tissue infiltration that occur after denervation.

## 2 Materials and methods

This prospective study consisted of 36 patients who had been referred to the laboratory of Clinical Neurophysiology at Tampere University Hospital for EDX examination due to lower limb symptoms or radiating lower back pain. The EDX examination was performed as part of normal patient care. The EDX studies were made using Cadwell Sierra Summit Electrodiagnostic Solution (Cadwell Industries Inc., Kennewick, WA, USA). The main reasons for referral and final EDX diagnoses are presented in Table 1. The patients also volunteered for additional HRUS and SWE studies of the TA and GCM muscles. The age, height, and weight of the patients were also recorded. The patients were then asked to subjectively assess whether they had any weakness in the studied muscles and, if so, for how many months the symptoms had persisted. All examinations were made during a single visit. Written informed consent was obtained from all participants. The Ethical Committee of Tampere University Hospital, Tampere, Finland approved the study. The study complied with the 2013 update of the declaration of Helsinki.

**Table 1:** The number of suspected conditions (reasons for referral) and the final assessed diagnosis based on the performed EDX (EDX findings). A patient might have two suspected conditions or findings in EDX. The patients in the group “Other” were referred due to cramps, suspected motor neuron disease, or unspecified lower extremity symptoms. For the final diagnoses, the “Other” group had focal fibular nerve neuropathies, neuromyotonia, and one case of late-onset spinal motor neuronopathy (LOSMoN).

	Radiculopathy	Polyneuropathy	Other	Normal
Suspected condition	21 (51 %)	10 (24 %)	10 (24 %)	
EDX findings	14 (38 %)	4 (11 %)	4 (11 %)	15 (40 %)

### 2.1 Nerve conduction studies

Sensory nerve conduction study (NCS) was performed with surface electrodes on the superficial fibular nerve and the sural nerve with a distance of 140 mm between the stimulating and recording electrode. Sensory nerve conduction velocities and amplitudes were used for the clinical diagnosis. However, as the focus of the study was on muscle diagnostics, they were not documented in the study. Motor NCS was performed on the fibular and tibial nerves. The compound muscle action potential (CMAP) amplitudes were recorded with surface electrodes from the extensor digitorum brevis (EDB) muscle for the fibular nerve/ L5 nerve root and from the abductor hallucis (AH) muscle for the tibial nerve/ S1 nerve root. Both nerves were stimulated with supramaximal stimulation intensity at the ankle at a distance of 80 mm from the recording electrode. The CMAP amplitudes were included in the analysis. Age-corrected reference values were used for the determination of normality.

### 2.2 Needle electromyography

Needle electromyography (EMG) was performed on 1 to 2 muscles per myotome from myotomes L4 to S1, innervated by different peripheral nerves in supine position. We used 30 HZ and 10,000 HZ filter settings. The patient was first asked to relax the examined muscle for the detection of insertion activity and spontaneous activity. Then, the motor unit potentials (MUPs) were assessed in slight voluntary contraction. Finally, the interference pattern (IP) was assessed in forceful voluntary contraction.

Neurogenic lesion was defined in EMG as incomplete IP with either changes in MUP size (larger units) or shape (longer duration of units, polyphasic units). The IP was graded as follows: 0 = normal; 1 = slightly reduced; 2 = moderately reduced; 3 = severely reduced. The presence of abnormal spontaneous activity in the form of fibrillations or positive sharp waves (FPs) was also noted and graded as follows: 0 = no FPs; 1 = single FPs in at least two insertions; 2 = moderate number of FPs in at least three insertions; 3 = several FPs in all insertions (Daube and Rubin, 2009; Rubin, 2012). The EMG results of the TA and GCM muscles were included in the analysis.

### 2.3 HRUS and SWE assessment

**Fig. 1:**
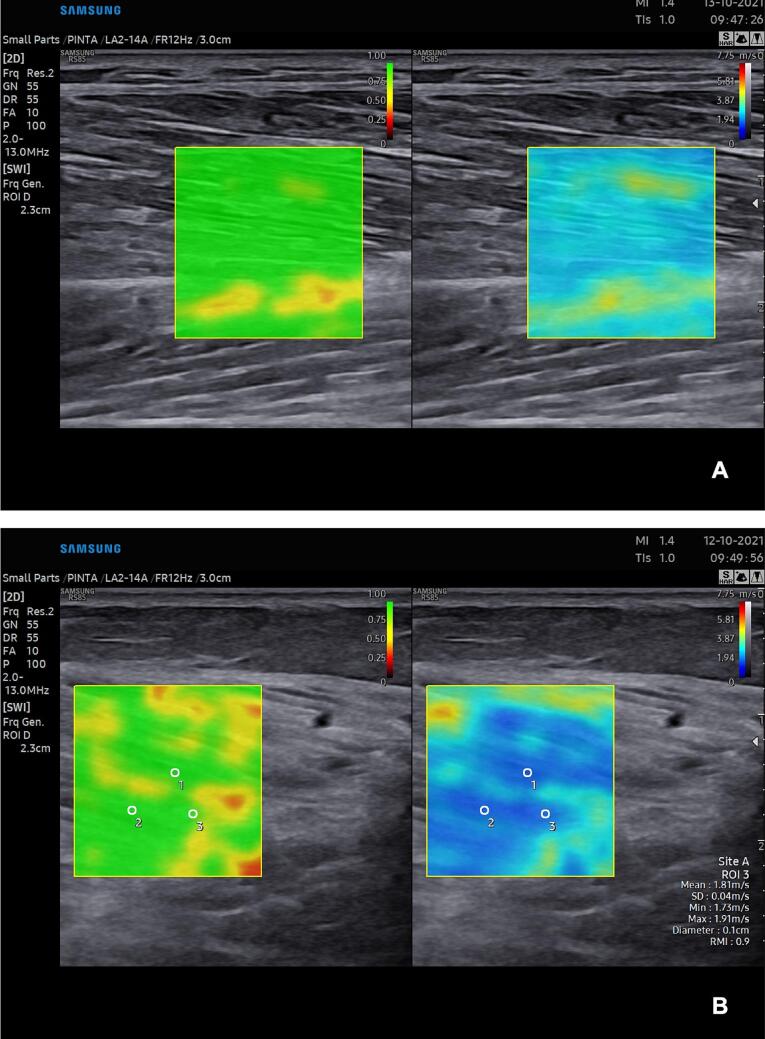
(A) SWE of a healthy Tibialis anterior muscle and (B) HS 2 scored Gastrocnemius medialis muscle in longitudinal orientation with three ROI circles in place. In both images, the reliability measurement index (RMI) map that estimates the quality of the SWE signal is shown on the left and the SWE stiffness map is shown on the right. In the RMI map, green areas are closest to RMI value 1 and red to RMI value 0. The closer the RMI value is to 1, the more reliable is the SWE signal. In the stiffness map, the color scale presents softer areas in dark blue and stiffer areas in red.

As the depth of the target area may influence SWE measurements, the relatively superficial TA and GCM muscles were chosen for examination (Romano et al., 2021, Carpenter et al., 2015, Alrashed and Alfuraih, 2021). The HRUS and SWE studies of the TA and GCM muscles were made using the Samsung RS85 prestige ultrasound system (Samsung Medison Co. Ltd, Seoul, Republic of Korea) using LA2-14A linear transducer (frequency range of 2.0 MHz to 14.0 MHz). The RS85 system measures 2D shear wave propagation with S-Shearwave Imaging™ technology. The stiffness map is overlayed on a conventional B-mode image with a simultaneous dual image, indicating the reliability of the SWE map with a reliability measurement index (RMI) (Fig. 1). The gain and frequency settings were kept the same for all examined patients. Examination room temperature was kept above 20 °C, and patients were encouraged to relax during the examination. One of the authors (KM) who had four years’ experience of conventional neuromuscular ultrasound and three years’ experience of SWE performed all the HRUS and SWE studies. The HRUS and SWE studies were made blinded from the EDX results. The muscles were located using anatomical landmarks.

**Fig. 2:**
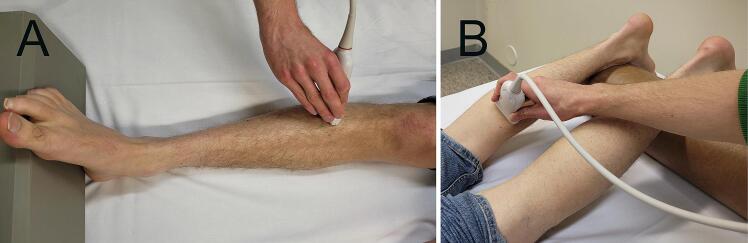
Demonstration of the experimental setup showing the topographical locations of the transducer on (A) the Tibialis anterior muscle, and (B) the Gastrocnemius medialis muscle.

In the HRUS assessment, the TA and GCM muscles were scanned in transverse orientation of the transducer in relation to the muscle fibers. The site where the echogenicity was evaluated was approximately the site where the needle was placed in EMG (Perotto et al., 2011). The topographical location of the transducer is shown in Fig. 2. Heckmatt score (HS) for each studied muscle was assessed from 1 to 4 using the Heckmatt grading scale. HS 1 represents the normal echogenicity of a heathy muscle in which the echogenicity of the muscle tissue is similar to that of the subcutaneous layer. HS 2–4 represent increased echogenicity with rising severity. In HS 2, the echogenicity is increased, but the bone echo under the muscle is clear. In HS 3, the echogenicity is increased, and the bone echo is reduced. In HS 4, the echogenicity is strongly increased, and the bone echo cannot be distinguished (Heckmatt et al., 1982).

The SWE was performed after the HRUS assessment. As it is known that limb muscle stretching or tension can influence SWE measurement (Romano et al., 2021, Rugel et al., 2020), care was taken to control the muscle length by controlling the joint position. For the TA muscle, the foot was stabilized to a 90° extension from the ankle, with the patient lying supine. For the GCM muscle, stabilization was removed and the patient turned to prone position. Support was adjusted under the ankles to achieve a small flexion of the knee. The ankle joint was in a relaxed neutral position. The ultrasound transducer was handheld, and care was taken not to compress the muscle. Good contact with the skin was ensured with copious amounts of gel. Surface EMG was not used. The orientation of the transducer in relation to muscle fibers was confirmed on B-mode image. Both the TA and GCM muscles were examined from two different sites approximately 10 cm apart in the proximal–distal direction. After denervation the replacement of muscle tissue with fibrosis and fat may be regional. Therefore, it is meaningful to measure the SWV at multiple sites. The proximal site was approximately the site where the needle was placed in EMG (Perotto et al., 2011). The topographical location of the transducer at the proximal site is demonstrated in Fig. 2. The SWE stiffness map was frozen, and three ROIs were inserted into the map at a depth of 1–2 cm. The location of each ROI was selected using the RMI, which had to be ≥ 0.7 for the location to be chosen. Intramuscular fibrous septa seen in the stiffness map were also avoided. The diameter of the ROI was 3 mm, which was chosen according to a previous study (Vuorenmaa et al., 2022). The average of the 3 ROIs from both measuring points were then calculated and recorded for the statistical analyses in both longitudinal (SWE-L) and transverse (SWE-T) orientation in relation to the muscle fibers. A variable (SWE-D) was calculated from the difference between these two measurements [SWE-D = SWE-L − SWE-T].

### 2.4 Statistical analyses

Statistical analyses were performed using IBM SPSS version 28.0 (SPSS Inc., Chicago, IL, USA). Data from the SWE were compared with the EDX results and HS. The Mann-Whitney *U* test was used to compare the differences between two independent groups and Kruskal-Wallis test for three or more groups. Correlations were tested with Spearman’s rho tests. In the Kruskal-Wallis multiple testing, the analyses were Bonferroni corrected. A summary of the variables evaluated in this study is presented in Table 2.

**Table 2:** Summary of variables evaluated in the study.

Method	Variable name	Explanation
Neve conduction study	EDB CMAP	Amplitude of compound muscle action potential recorded from extensor digitorum brevis muscle
	AH CMAP	Amplitude of compound muscle action potential recorded from abductor hallucis muscle

Electromyography*	FPs	Fibrillations or positive sharp waves
	IP	Interference pattern

High resolution ultrasound*	HS	Heckmatt score to evaluate muscle echogenicity

Shear wave elastography*	SWE-L	Shear wave velocity measured in longitudinal orientation
	SWE-T	Shear wave velocity measured in transverse orientation
	SWE-D	Calculated difference between SWE-L and SWE-T
		Other gathered information on patients:
		− Age, sex, height, weight, BMI
		−Subjective assessment of Tibialis anterior and Gastrocnemius muscle weakness
		−Duration of possible muscle weakness

* Electromyography, HRUS and SWE were performed on the Tibialis anterior and Gastrocnemius medialis muscles.

## 3 Results

There were 16 male and 20 female patients. Patient demographics are presented in Table 3. Most patients underwent bilateral EDX examination. In a few cases, however, asymptomatic limbs were only partly examined for side-to-side comparison. In the NCS studies, 62 EDB CMAPs and 61 AH CMAPs were recorded. Needle EMG was performed on a total of 68 TA and 66 GCM muscles of 36 patients, in which neurogenic injury was noted in 76 % of the examined TA muscles and in 55 % of the GCM muscles. HRUS and SWE were performed bilaterally in all patients, except for 4 GCM muscles from two patients. These patients had more than 2 cm subcutis over the GCM muscle, preventing the ROI from being set at a depth of 1–2 cm.

**Table 3:** Mean, range, and standard deviation of patient age, height, weight, and duration of subjective muscle weakness.

	Mean	Range	Standard deviation
Age [years]	58	29–85	14.2
Height [cm]	170	151–189	8.8
Weight [kg]	80	45–135	19.6
BMI	27	18–38	5.6
Duration of weakness [months]	34	0–120	38.3

### 3.1 Association of EDX and US findings with patient demographics

Amongst muscles with normal EMG findings, patient age or BMI did not correlate with the SWE-D in either muscle. However, in GCM muscles, SWE-D correlated negatively with weight (p = 0.017, r = -0.263). Furthermore, there was no significant difference between men and women in SWE-D in either muscle. In both muscles, the HS correlated positively with patient age (p < 0.001 for both muscles, TA r = 0.429 and GCM r = 0.406). HS did not correlate to patient BMI and no statistical difference in the distribution of the HS groups between men and women in either muscle was found.

Amongst muscles with neurogenic findings in EMG, the duration of the subjective muscle weakness symptoms did not correlate with the SWE parameters in either muscle. However, the duration of the subjective muscle weakness symptoms correlated positively with HS in the GCM muscles (p = 0.003, r = 0.464), but not in the TA muscles. In the TA muscles, the duration of the weakness correlated positively with the amount of IP reduction (p = 0.028, r = 0.349) and negatively with the EDB CMAP amplitude (p = 0.032, r = -0.359). In the GCM muscles, the duration of the weakness correlated positively with the amount of IP reduction (p < 0.001, r = 0.716) and with the quantity of FPs (p = 0.001, r = 0.488).

### 3.2 Associations between the EDX and SWE

The correlation coefficient and p-values for the correlations between the EMG and SWE parameters are presented in Table 4. In the TA muscles, SWE-D correlated negatively with the quantity of FPs and the amount of IP reduction. SWE-T did not correlate significantly with the quantity of FPs, nor with the reduction of IP. SWE-L correlated negatively with IP reduction. However, there were no significant correlation with the number of FPs. The EDB CMAP amplitude did not correlate with the SWE parameters.

**Table 4:** The p-values and correlation coefficient values for the correlations between the SWE parameters and IP, FP and HS (Spearman’s rho tests). TA: Tibialis anterior, GCM: Gastrocnemius medialis, IP: Interference pattern, FP: Fibrillations or positive sharp waves, HS: Heckmatt score, n.s.: Non-significant (p > 0.05).

	IP		FP		HS	
	*p*	*r*	*p*	*r*	*p*	*r*
**TA**						
SWE-D	0.006	−0.236	0.032	−0.185	<0.001	−0.360
SWE-L	<0.001	−0.294	n.s.		<0.001	−0.320
SWE-T	n.s.		n.s.		0.007	−0.228

**GCM**						
SWE-D	0.030*	−0.285*	n.s.		<0.001	−0.299
SWE-L	n.s.		n.s.		0.002	−0.259
SWE-T	n.s.		n.s		n.s.	

* in the normal weight group.

In the GCM muscles, the EMG variables did not correlate with any of the SWE parameters in the total study material. However, there was a positive correlation between the AH CMAP amplitude and SWE-D (p = 0.021, r = 0.210).

As the SWE-D correlated with patient weight, the patients were divided into two groups according to BMI: ≤ 25 (normal weight, N = 15 patients) and > 25 (obese, N = 19 patients). There was a significant difference in the overall SWE-D values between patients with normal weight (mean 0.667 m/s, std ± 0.560) and obese patients (mean 0.395 m/s, std ± 0.592) in the GCM muscles (p = 0.007).

**Fig. 3:**
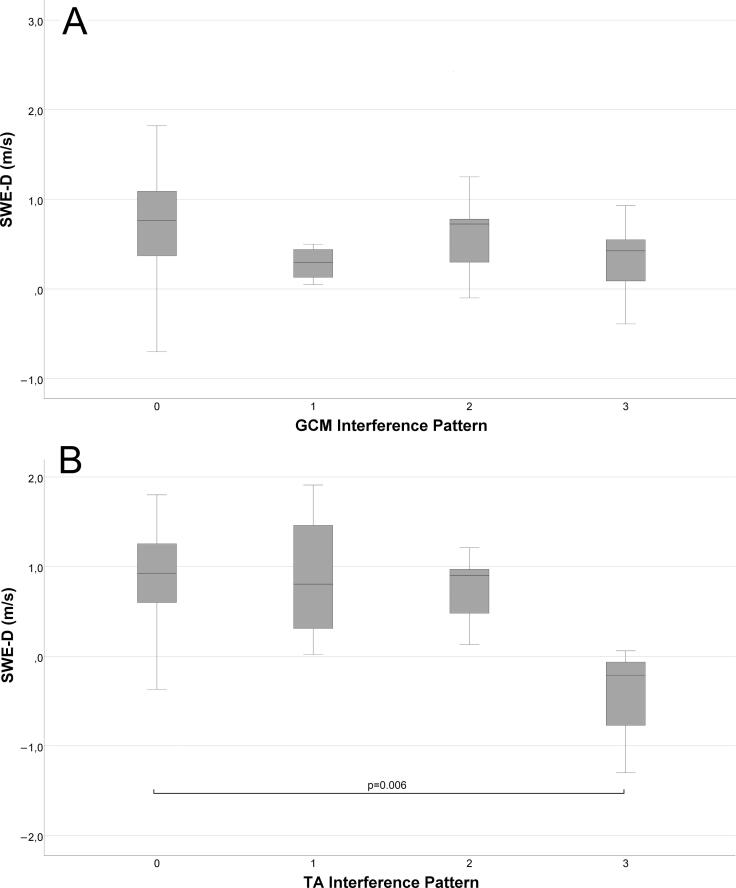
Boxplot showing the SWE-D values according to the categories of the reduced interference pattern (IP) among patients with normal BMI (≤ 25) in A) the Gastrocnemius medialis (GCM) muscles and B) in the Tibialis anterior (TA) muscles. The line within the boxes shows the mean of SWE-D for each IP group. The borders of the box show the upper and lower quartile and the whiskers the maximum and minimum SWE-D value. The Bonferroni corrected p-value for a significant difference between IP categories 0 and 3 in TA muscles is presented above the line.

Further, in the group of patients with normal BMI, a significant difference in the SWE-D between groups of normal EMG (mean 0.754 m/s, std ± 0.594) and abnormal EMG (mean 0.428 m/s, std ± 0.410) was found (p = 0.025). In the group of obese patients, however, this significance was lost. There was also a negative correlation between the SWE-D and the amount of IP reduction in the normal weight group (p = 0.030, r = -0.285, Fig. 3). However, there was no correlation in the obese group. SWE-T or SWE-L did not correlate with IP reduction in any of the subgroups in the GCM muscles.

Furthermore, in the TA muscles, when the obese patients were filtered out, the correlation coefficient between the SWE-D and the amount of IP reduction (p = 0.016, r = -0.310, Fig. 3) and the FPs (p = 0.021, r = -0.298) strengthened slightly. The SWE-D, SWE-L and SWE-T values for both muscles according to the IP categories are shown in Supplementary Table S1.

### 3.3 Associations between the HS and SWE

The correlation coefficient and p-values for the correlations between the HS and SWE parameters are presented in Table 4. The HS correlated negatively with SWE-D and SWE-L in both the TA and the GCM muscles. The SWE-T correlated positively with the HS in the TA muscles, but not in the GCM muscles.

**Fig. 4:**
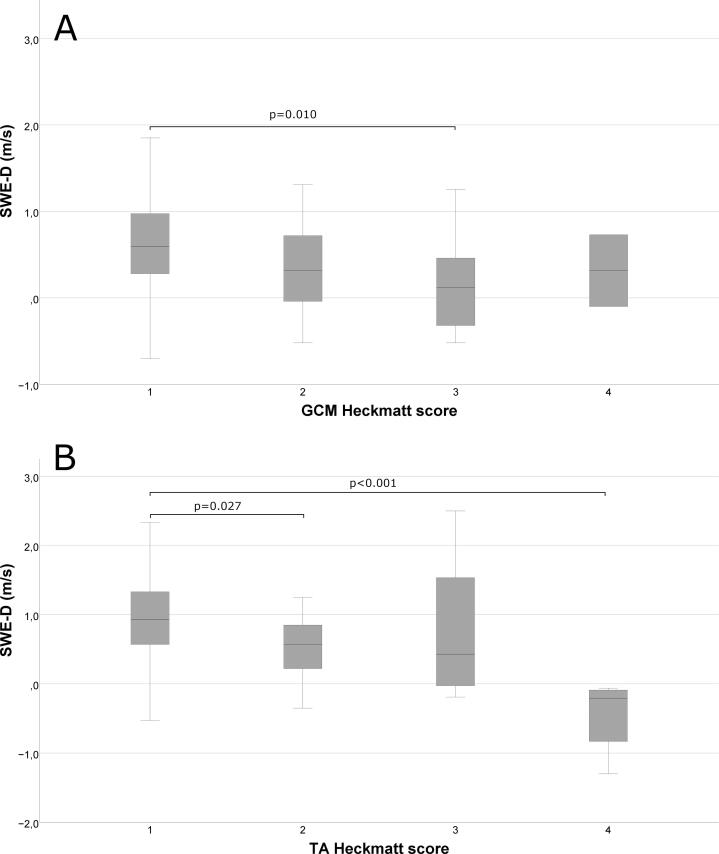
Boxplots showing the SWE-D values according to the Heckmatt score (HS) in A) the GCM muscles and B) in the TA muscles. The HS groups that had significantly different SWE-D values compared to the other HS groups are marked with a horizontal line. The Bonferroni corrected p-value for each significant difference is presented above the line. The line within the boxes shows the mean of SWE-D for each HS group. The borders of the box show the upper and lower quartile and the whiskers the maximum and minimum SWE-D value.

In both muscles, there were significant differences in SWE-D between different HS categories (p < 0.001 for the TA muscle and p = 0.007 for the GCM muscle). The boxplots in Fig. 4 demonstrate the differences between the groups.

## 4 Discussion

As the main result of this study, we can present a novel SWE-based method to evaluate muscle denervation. Loss of the normal arrangement of muscle fibers leads to a converging of the longitudinal and transverse SWV. In the present study, this phenomenon was measured by the SWE-D variable, which was calculated from the difference in longitudinal and transverse SWV. SWE-D correlated negatively with the number of fibrillation potentials in the TA muscles and to the degree of reduction of interference pattern in the TA muscles and in the GCM muscles in the normal weight group. The degree of reduction of interference pattern is usually thought to reflect the severity of denervation. The SWE-D was also associated negatively with increased echogenicity, evaluated with the Heckmatt grading scale.

The time-dependent effects of muscle histopathology seem to be related to SWE. Muscle histopathology is dependent on the age of the lesion (Gonzalez et al., 2017, Tews et al., 1994, Joe et al., 2010, Ehmsen and Ahmet, 2020), and this time-dependent effect has previously been shown to be detectable from muscle SWE (Rosskopf et al, 2016). For example, Rosskopf et al. (2016) found that after supraspinatus muscle injury the SWV tends to first decrease with increasing fatty infiltration and then slightly increases in the final stage. In their study, they measured the SWV in transverse orientation in relation to the muscle fibers. This may also relate to our findings, where there was no linear correlation between the number of FPs or IP reduction and the SWE-T. Furthermore, the degree of the neurogenic lesion was associated with the duration of the subjective weakness of the studied muscle, but not with the SWE-D. This also suggests that SWE-D is independent from the changing histopathology. However, to verify this assumption, the histopathological changes should be studied microscopically from muscle biopsies after SWE of the site of the biopsy. The SWE-L seems to partially associate with the severity of the neurogenic lesion and reflects the loss of anisotropy, as it correlates with the amount of IP reduction in the TA muscles but not with the quantity of FPs.

The accuracy of SWE to detect denervation may depend on the studied muscle. In the entire study material, the SWE-D and the EMG parameters were more often associated in the TA muscles than in the GCM muscles. The TA is a superficial muscle with a low degree of pennation and with lower levels of adipose tissue covering it than the GCM muscle. Also, in the previous literature it has been shown that there is lower variability in SWE measurements in the TA muscle than in the GCM (Romano et al., 2021). On the other hand, it could be speculated, that also neurogenic injury might lead to a different kind of fatty infiltration in these different muscles, as there is a marked difference in muscle morphology and arrangement of innervation (Wolf and Kim,1997). Furthermore, joint positions, and therefore muscle length and prestress of the muscle, also affect on SWE (Romano et al., 2021, Rugel et al., 2020). In the present study exact knee and ankle angels were not measured, which may have led to small variations of joint angles between patients and affected the acquired SWV values.

Obesity must be acknowledged in the SWE-based assessment of muscle denervation. In the GCM muscles, the SWE-D correlated negatively with patient weight. In these muscles, SWE-D was associated with muscle denervation only among patients with normal BMI. Thus, in both conditions, obesity and muscle denervation, the SWE-D decreases compared to healthy muscle tissue. Moreover, it has been shown that muscle fat infiltration may be remarkable in obese patients. As many as twice the number of fat droplets has been seen in the muscles of obese individuals compared to individuals of a normal weight (Malenfant et al., 2001). Thus, among patients with obesity, care should be taken in the interpretation of reduced SWE-D, since the reduction may be caused by a normal fatty infiltration related to obesity instead of fatty infiltration related to denervation. This should also be considered in future studies.

When the Heckmatt grading scale and SWE-D are compared as methods of assessing muscle denervation, the use of the Heckmatt grading scale is not uncomplicated either. It is known that aging as well as obesity causes the enhanced echogenicity of muscle tissue (Pereira et al., 2021). The benefit of SWE-D compared to the Heckmatt grading scale may be that SWE-D does not seem to be age dependent; however, this finding still needs further study, as the number of patients in our study was rather small. Side-to-side comparison would be beneficial in cases where patients have unilateral symptoms. Furthermore, such a comparison could provide an internal reference for the Heckmatt grading scale as well as the SWE-D value. Objectivity and quantitativeness are advantages of SWE-D when compared to the Heckmatt grading scale.

This study has several limitations that should be considered. First, although the patient baseline characteristics were broadly distributed, the number of participants in the study was quite small. Therefore, this study should be considered merely as a pilot study. Second, surface EMG was not used simultaneously with the SWE study, leaving a small chance that some muscles were not completely relaxed, even if muscle relaxation was encouraged and the limbs supported during the measurements. Third, the correlation coefficients of SWE-D presented are only slight to modest. Of the two studied EMG parameters, the amount of IP reduction correlated slightly stronger with the SWE-D than the FPs. This is most likely because the FPs relate to acute or subacute denervation in which the myofibrillar rearrangement and disruption may not yet have occurred to a broad extent. The IP represents a more stable state of chronic denervation. Therefore, although the results are promising, the SWE-D should not yet be used solely in the diagnostics of neurogenic injury, and EDX remains the optimal tool. It must also be kept in mind that the change towards isotropy is not specific to denervation and can also be seen, for example, in myopathies (Albayda and van Alfen, 2020). Fourth, the assessment of EMG is subjective, even though it has been found to be clinically reliable and reasonably reproducible (Daube and Rubin, 2009). Quantitative motor unit potential analysis could have been performed to enhance accuracy. Also, the assessment of the Heckmatt grading scale is partly subjective, and quantitative grayscale analysis or echovariance measures would have been more appropriate.

## 5 Conclusion

In conclusion, the findings of this study show that SWE is a promising application for quantifying muscle denervation by using it as a marker of the loss of muscle anisotropy. However, EMG remains the optimal tool for determining muscle denervation and its severity. The findings of this study should be verified in a larger material, and the effect of obesity on SWE should be further investigated. In future studies, the function of SWE-D in myopathies should also be assessed.

SWE is a rather new ultrasound-based application and has potential as a complementary neuromuscular ultrasound study. However, before SWE can be used in clinical practice, the open questions need to be answered in future research.

## 6 Statement of ethics

Data are not available due to legal restrictions. Supporting data are not available.

This study was financially supported by Competitive State Research Financing of the Expert Responsibility area of Tampere University Hospital (Grant 9AB008,). Competitive State Research Financing of the Expert Responsibility area of Tampere University Hospital did not have any involvement in the study design, data collection, analysis or interpretation of the data, or in the writing of the report and publication process.

Written informed consent was obtained from all participants.

The Ethical Committee of Tampere University Hospital (reference number R19002; date of approval 19.2.2019) has approved this study.

## Declaration of competing interest

The authors declare that they have no known competing financial interests or personal relationships that could have appeared to influence the work reported in this paper.
