# Supplementary material for: Shear wave elastography as a marker of anisotropy in denervated muscle tissue
Source: Clin Neurophysiol Pract. 2025 Mar 5;10:95–103. doi: 10.1016/j.cnp.2025.02.007 (PMC11951941; doi:10.1016/j.cnp.2025.02.007)
Supplement: Supplementary Data 1 [file mmc1.docx]

| **Tibialis anterior** | | | | | | | | | |
| --- | --- | --- | --- | --- | --- | --- | --- | --- | --- |
|  | **SWE-D (m/s)** | | | **SWE-L (m/s)** | | | **SWE-T (m/s)** | | |
| **IP category** | Mean | Min.-Max. | Std. Dev. | Mean | Min.-Max. | Std. Dev. | Mean | Min.-Max. | Std. Dev. |
| 0 | 0.92 | -0.53–2.33 | ± 0.52 | 2.83 | 1.95–3.95 | ± 0.39 | 1.92 | 1.30–3.94 | ± 0.39 |
| 1 | 0.52 | -0.83–1.95 | ± 0.72 | 2.55 | 2.09–3.20 | ± 0.38 | 2.03 | 1.20–3.05 | ± 0.51 |
| 2 | 0.72 | -0.11–1.44 | ± 0.45 | 2.57 | 2.05–3.13 | ± 0.28 | 1.85 | 1.22–2.96 | ± 0.44 |
| 3 | 0.41 | -1.30–2.50 | ±1.40 | 2.86 | 2.02–4.37 | ± 0.87 | 2.45 | 1.64–3.44 | ± 0.67 |
| All patients | 0.80 | -1.30–2.50 | ±3.80 | 2.74 | 1.95–4.37 | ± 0.42 | 1.95 | 1.20–3.94 | ± 0.45 |

| **Gastrocnemius medialis** | | | | | | | | | |
| --- | --- | --- | --- | --- | --- | --- | --- | --- | --- |
|  | **SWE-D (m/s)** | | | **SWE-L (m/s)** | | | **SWE-T (m/s)** | | |
| **IP category** | Mean | Min.-Max. | Std. Dev. | Mean | Min.-Max. | Std. Dev. | Mean | Min.-Max. | Std. Dev. |
| 0 | 0.56 | -0.70–2.38 | ± 0.62 | 2.57 | 1.75–4.31 | ± 0.44 | 2.00 | 1.31–3.06 | ± 0.44 |
| 1 | 0.49 | -0.32–1.77 | ± 0.55 | 2.40 | 2.04–3.06 | ± 0.39 | 1.90 | 1.29–2.36 | ± 0.34 |
| 2 | 0.43 | -0.48–1.25 | ± 0.46 | 2.56 | 2.20–3.23 | ± 0.30 | 2.12 | 1.70–2.68 | ±0.34 |
| 3 | 0.14 | -0.52–0.93 | ± 0.53 | 2.19 | 1.66–2.90 | ± 0.38 | 2.05 | 1.57–2.75 | ±0.44 |
| All patients | 0.51 | -0.70–2.38 | ± 0.60 | 2.52 | 1.66–4.31 | ± 0.42 | 2.01 | 1.29–3.06 | ±0.42 |

Supplementary Table S1. SWE-D, SWE-L and SWE-T velocity (m/s) values according to the degree of reduction of interference pattern (IP) in Tibialis anterior and Gastrocnemius medialis muscles. Min.-Max.: sample minimum and maximum, Std. Dev.: Standard deviation.
